# The road beyond licensing: the impact of a driver licensing support program on employment outcomes for Aboriginal and Torres Strait Islander Australians

**DOI:** 10.1186/s12889-021-12218-1

**Published:** 2021-11-23

**Authors:** Bobby Porykali, Patricia Cullen, Kate Hunter, Kris Rogers, Melissa Kang, Nareen Young, Teresa Senserrick, Kathleen Clapham, Rebecca Ivers

**Affiliations:** 1 School of Public Health, University of Technology Sydney, 15 Broadway, Ultimo, NSW 2007 Australia; 2 The George Institute for Global Health, Level 5, 1 King Street, Newtown, NSW 2042 Australia; 3 School of Population Health, University of New South Wales, Sydney, NSW 2052 Australia; 4 Ngarruwan Ngadju, First Peoples Health and Wellbeing Research Centre, University of Wollongong, Wollongong, NSW 2522 Australia; 5 Queensland University of Technology, 130 Victoria Park Road, Kelvin Grove, QLD 4059 Australia

**Keywords:** Aboriginal, Torres Strait Islander, Employment, Driver licensing support program, Driver licence

## Abstract

**Background:**

With increasingly tough graduated driver licensing laws in all Australian States and Territories, driver licensing support programs are recognised as being important to support Aboriginal and Torres Strait Islander peoples to obtain a driver licence. Such programs appear to improve licensing attainment rates, but few studies have examined the broader impact that these programs can have. This research aims to 1) examine the impact of a New South Wales (NSW) based driver licensing support program (Driving Change) on client employment outcomes; 2) assess the influence of geographical area of program delivery on driver licence attainment.

**Methods:**

Driving Change was delivered from February 2013 to August 2016 in 4 urban and 7 regional Aboriginal communities of NSW. Clients were followed-up at 6 months or more following contact with the program as part of routine program operations. Descriptive statistics and regression models were used to analyse data.

**Results:**

From 933 clients contacted 254 agreed to provide feedback, a response rate of 27%. Those that responded were mostly female (57%), aged 24 years and under (72%), unemployed (85%) with secondary education or less (71%) and from a regional area (74%).

Adjusted logistic regression indicated that clients who achieved an independent licence were more likely (OR: 2.5, 95% CI: 1.22–5.24, *p* = 0.011) of reporting a new job or change in job than those who did not attain a licence. Clients from regional areas were more likely (OR: 1.72, 95% CI: 1.27–2.33, *p* < 0.001) to gain an independent licence than those from urban areas. There was no difference in employment outcomes (OR: 1.2, 95% CI: 0.53–2.52, *p* = 0.719) for clients from urban compared to regional areas.

**Conclusion:**

The Driving Change program appears to be effective in improving employment outcomes for those who gained a licence. Clients from regional areas were more likely to gain a licence compared to those in urban settings, and were predominantly young and unemployed, often a hard to reach cohort. Future licensing programs being delivered in regional areas need integrated pathways into employment opportunities to provide holistic services that address the social and economic challenges faced by Aboriginal and Torres Strait Islander Australians.

## Background

Australia has a vast land mass. With its population widely spread across major cities and regional and remote towns, access to public or private transport is necessary for everyday life. Where there is an inability to access public or private transportation individuals experience transport disadvantage. In regional and remote towns of Australia compared to major cities there are fewer public transportations options, therefore having a valid driver licence and access to private transport is essential for mobility, social inclusion and participation in critical activities such as employment [1, 2]. These geographical areas are often further associated with profiles of lower socioeconomic status and lower levels of average household income which are known barriers to obtaining a driver licence and to ownership of a private vehicle [1]. Higher proportions of Aboriginal and Torres Strait Islander First Nations Australians reside in regional and remote areas than do non-Indigenous Australians [3]. Although poor data precludes robust estimates, the percentage of licence holders amongst eligible Aboriginal and Torres Strait Islander population aged 16 years and above is considerably lower than 70%, which is the observed rate amongst eligible non-Indigenous Australians [2, 4]. Transport disadvantage can therefore disproportionately impact Aboriginal and Torres Strait Islander Australians and contribute to a cycle of licensing adversity [5], whereby people who have no licence have limited access to health facilities, job options and study opportunities [1].

Culturally relevant driver licensing programs have been delivered with increasing frequency over the past 10 years to support Aboriginal and Torres Strait Islander peoples to obtain a driver licence, however very few of these programs have been evaluated [6, 7]. The few evaluated programs have shown an increase in licensing attainment amongst program clients [8–11]. These programs are predominantly delivered in regional and remote settings where transport disadvantage is most pronounced, and employment rates and education levels are lowest [12, 13]. For Aboriginal and Torres Strait Islander peoples, employment rates vary considerably by geographical area lived compared to non-Indigenous Australians. For instance, employment rates for Aboriginal and Torres Strait Islander people are highest in major cities (54.1% for Aboriginal and Torres Strait Islander people compared with 71.8% for non-Indigenous Australians), followed by inner regional (47.2% versus 70.7%), outer regional areas (43.5% versus 72.3%) and lowest in remote (39.8% versus 79.3%) and very remote areas (30.8% versus 85.3%) [13].

Employment is a strong social determinant for improving Aboriginal and Torres Strait Islander people’s health outcomes [14, 15] and those who are economically advantaged are more likely to have lower prevalence of health risk factors [16]. Though employment rates have been on the rise over the last decade from 48.2 to 49.1% between 2008 and 2018–2019, there is still much needed improvements to reach parity with the 75% employment rates achieved by the labour force (age 15–64 years) of non-Indigenous Australians [17]. It is known that higher levels of education and training are important determinants for reaching parity in employment outcomes [18], but studies have also shown from the perspectives of Aboriginal and Torres Strait Islander young peoples the importance placed on driving as being an enabler to gaining employment and accessing job opportunities [19]. Our past research also showed that having a licence is associated with increased likelihood of having a job [2]. Despite the emerging evidence limited research beyond this has been placed on further exploring the broader impact that these programs have beyond licence attainment.

In summary, a high proportion of Aboriginal and Torres Strait Islander peoples reside in regional and remote areas [3], where they experience the lowest rates of employment [13], where transport disadvantage is most pronounced and where licensing programs are being delivered with increased frequency [7]. The purpose of this paper is to 1) examine the impact that driver licensing programs have on client employment outcomes; 2) assess the influence of geographical area of program delivery on driver licence attainment. This will be achieved through post program data analysis of a New South Wales (NSW) based Aboriginal and Torres Strait Islander driver licensing support program - Driving Change.

### The program

Driving Change was a culturally relevant, end-to-end driver licensing program that provided support to Aboriginal and Torres Strait Islander peoples to navigate through the NSW Government’s graduated licensing system and obtain a driver licence.

Informed development of this culturally relevant program was underpinned by extensive community consultation, and formative evaluation [20, 21]. This included a pilot survey in a remote north-western NSW Aboriginal community at Bourke Aboriginal Medical Service [22], then a large observational study involving four Aboriginal community-controlled health services [23]. Stakeholder interviews were also conducted with a range of policy makers and key community contacts to understand both barriers to licensing, licensing rates and impact of licensing [2]. As a result, the Driving Change program was developed and implemented in a way acceptable to clients and stakeholders [21]. Governance of the program included consistent participation from Government stakeholders, through involvement of the Transport for NSW Centre for Road Safety (TfNSW CRS) and Roads and Maritime Services (Aboriginal programs) in the programs steering committee.

The program was delivered from February 2013 to August 2016 widely across the State of NSW in 11 Aboriginal communities of: Kempsey, Taree, Raymond Terrace, Redfern, Campbelltown, Shell harbour, Dubbo, Dareton, Condobolin, Griffith and Wagga Wagga [8]. The program facilitated access to local services, intensive case management, providing mentoring for young people through the licensing system and provided support to address licensing sanctions imposed by the State [8].

## Method

### Design

This study is a quantitative analysis of post program client interview data.

### Processes and measurements

Data was collected as a part of the program’s quality assurance processes; clients who attended the program were systematically followed up between June 2014 – August 2016.

### Baseline data

Upon initial contact with the program, baseline client data collected contained information on gender (male, female), age (16–24 and 25+ years), employment status (employed, unemployed), education (secondary educated or less, tertiary educated) and community of program delivery, as well as reasons clients were accessing the program and expectations.

### Follow up data

As part of routine program operations, clients were contacted by phone 6 months or more after their initial contact with the program and invited to participate in answering a set of open-ended post-program questions to ascertain: the levels of satisfaction with the program, services and support accessed; licensing outcomes; additional program outcomes reported for employment, education, family responsibilities and/or social inclusion. The specific outcomes of interest for analysis were:

- “Whether the client had a change in licence type as a result of the program”
- “Whether since joining the program the client has had a change in work, and if yes to specify employment change (‘unemployed and now employed’ or ‘change jobs’)”

The 11 communities where the program was delivered were stratified as urban areas or regional areas - regional areas encompassed inner regional, outer regional, remote and very remote. Stratification of communities as urban (Raymond Terrace, Redfern, Campbelltown and Shellharbour) or regional (Kempsey, Taree, Dubbo, Dareton, Condobolin, Griffith and Wagga Wagga) was in accordance with the classification of the Australian Bureau of Statistics Remoteness of Areas [24].

### Analyses

Descriptive statistics (frequency and percentage distributions) were performed to determine respondents’ characteristics.

Logistic regression models were used to estimate odds ratio of:

a) The association between licensing attainment and self-reported improvement in employment outcomes;
  b) Adjustment for age, gender and carer responsibilities; these were considered important as younger clients were more likely to be engaged in educational commitments, with females and carers more likely to have family priorities impacting on employment outcomes.
a) The association between geographical area on licensing attainment outcomes;
  b) Adjustment for literacy, household licensing status, level of contact with the program, age group and level of needs; as these variables influence the ability of a client to successfully obtain a licence.
a) The association between geographical area and self-reported improvement in employment opportunities;
  b) Adjustment for age, gender and carer responsibilities; as these variables were considered to impact most on employment outcomes.

### Ethics approval

All methods were carried out in accordance with the relevant guidelines and regulations, and all experimental protocols were approved by the Aboriginal Health and Medical Research Council Human Ethics Committee of New South Wales (Eth: 964/13). Informed consent was obtained from all subjects and/or their legal guardian(s). Data was analysed using Statistical Analysis System (SAS) version 9.4 (SAS Institute, Cary, NC, USA).

## Results

Nine hundred and thirty-three people enrolled in the Driving Change program, provided baseline data and were invited to participate in the 6-month follow up interview. A total of 254 clients out of 933 participated in answering the quality assurance post program questions, a response rate of 27%. The majority of those who responded were female, aged 24 years and under, unemployed, with secondary education or less and from a regional area at baseline (Table 1). Two key differences were observed at baseline between respondents (*n* = 254) and non-respondents (*n* = 679) – a higher proportion of respondents were employed (15% versus 9%) and tertiary educated (29% versus 20%) than non-respondents. Of the respondents, those reported gaining a job/or having a change in job proportionally were more often male, under the age of 24 years, secondary educated or less and resided in regional areas (Table 2).

**Table 1:** Comparison of program client characteristics (respondents versus non-respondents)

	Respondents	Non-Respondents	Program clients
***N (%)***	*254 (27)*	*679 (73)*	*933 (100)*
**Gender**			
Male	108 (43)	303 (45)	*411 (44)*
Female	146 (57)	376 (55)	*522 (56)*
**Age (years)**			
< 24	182 (72)	467 (69)	*646 (69)*
> 25	72 (28)	212 (31)	*287 (31)*
**Employment**			
Employed	38 (15)	61 (9)	*99 (11)*
Unemployed	216 (85)	618 (91)	*834 (89)*
**Education**			
≤ Secondary education	180 (71)	538 (79)	*718 (77)*
Tertiary educated	74 (29)	141 (21)	*215 (23)*
^**a**^**ROA**			
Urban	67 (26)	148 (22)	*215 (23)*
Regional	187 (74)	531 (78)	*718 (77)*

^a^Area classification according to ABS 2016 Remoteness of Area (ROA) [24]

**Table 2:** Respondent employment outcomes (gaining a job/or having a change versus unemployed/no change)

	Employment gained / changed	Unemployed / no change in employment
*N (%)*	*38 (15)*	*216 (85)*
**Gender**		
Male	23 (61)	85 (39)
Female	15 (39)	131 (61)
**Age (years)**		
< 24	25 (66)	157 (73)
> 25	13 (34)	59 (27)
**Education**		
≤ Secondary education	23 (61)	157 (73)
Tertiary educated	15 (39)	59 (27)
^**a**^**ROA**		
Urban	14 (37)	53 (25)
Regional	24 (63)	163 (75)

^a^Area classification according to ABS 2016 remoteness of area (ROA) [24]

Regression analyses highlighted in Table 3 indicted that clients who achieved an independent licence were more likely (OR: 2.6, 95% CI: 1.27–5.23, *p* = 0.007) to reporting gaining a job/or having a change in job. After adjustments were made for potential confounders (age, gender and carer responsibilities) the relationship still held with clients being more likely (OR: 2.5, CI: 1.22–5.24, *p* = 0.011) to report gaining a job/or having a change in job. For licensing attainment by geographical area, clients participating in the program from regional areas were more likely (OR: 1.43, 95% CI: 1.04–1.97, *p* = 0.028) to obtain an independent licence than those from urban areas. After adjustments were made for potential confounders (literacy, household licensing status, level of contact with the program, age group and level of needs) the relationship held with clients participating in the program from regional areas being more likely (OR: 1.72, 95% CI: 1.27–2.33, *p* <0.001,) to obtain an independent licence than those from urban areas. There were no differences in clients reporting having gained a job/or having a change in job between urban or regional areas (OR: 1.1, 95% CI: 0.52–2.39, *p* = 0.780,), including after adjusting for age, gender and carer responsibilities (OR: 1.2, 95% CI: 0.53 0 2.52, *p* = 0.719).

**Table 3:** Regression analysis

Model	Odds Ratio	Confidence Intervals (95%)	***p***-value
**1 a)**	2.6	1.27–5.23	0.007
**b)**	2.5	1.22–5.24	0.011
**2 a)**	1.43	1.04–1.97	0.028
**b)**	1.72	1.27–2.33	<0.001
**3 a)**	1.1	0.52–2.39	0.780
**b)**	1.2	0.53–2.52	0.719

## Discussion

This study showed the direct impact that driver licensing programs can have on improving employment outcomes for Aboriginal and Torres Strait Islander clients and highlighted the influence that geographical area of program delivery has on licence attainment. We found that respondents who obtained a driver licence as a result of participation in the Driving Change program were more likely (OR: 2.5, CI: 1.22–5.24, *p* = 0.011) to report gaining a job or having a change in jobs. Respondents from regional areas were more likely (OR: 1.72, 95% CI: 1.27–2.33, *p* < 0.001) to obtain a driver licence than those participating in the program from urban areas.

Health outcomes of an individual and their communities are closely linked to employment status, and the influence that this has on improving economic and social circumstances [14, 25]. Through post program follow-up, we highlighted a significant self-reported improvement in employment outcomes for clients who obtained a driver licence, and it was shown the majority (69%) of all clients who participated in the Driving Change program were under the age of 24 years. Young Aboriginal and Torres Strait Islander Australians between the ages of 16–24 years experience the highest rates of unemployment within their population [13]. Engaging these young people in programs that promote economic participation and social inclusion are important contributors to improving the health trajectory of the population [26]. The capacity displayed through this licensing program to capture a young cohort of the population who are unemployed serves as an opportune platform for continued engagement into employment pathways post licence attainment.

In existing efforts to target the younger aged population recommendations from the Australian Law Reform Commission (ALRC) 2018 indicated where Aboriginal and Torres Strait Islander young people are likely to complete secondary education and unlikely to face other identified obstacles (such as access to birth certificates), driver licence programs could constitute an elective in the school curriculum [4]. This does provide an opportunity to improve licensing rates amongst Aboriginal and Torres Strait Islander young peoples, but with any rise in numbers of young novice driver on the road there is also a risk of increased transport related crashes, injuries and deaths [23, 27]. To mitigate this risk, it is important that implementation of such culturally relevant driver licensing programs be tailored to employment requirements, for instance students wishing to pursue an apprenticeship and/or trade. Results from this analysis indicate that respondents who achieved improved employment outcomes were predominately under the age of 24 years (66%) and were secondary educated or less (61%). Therefore, capturing this young target demographic whilst engaged in education is important as research shows the longer an individual remains not employed, not undergoing education and/or training, the more likely they are to remain unemployed [18]. Additional areas of interest could be focussed on the benefit of using licensing programs amongst current initiatives for keeping Aboriginal and Torres Strait Islander youth engaged in secondary education.

The Driving Change program was delivered within both urban and regional settings, with sites chosen for program delivery based upon: communities need, engagement, and support of program; the population of Aboriginal and Torres Strait Islander residents; and socioeconomic status of the area. Research shows employment rates in urban areas for Aboriginal and Torres Strait Islander peoples who are tertiary educated are comparable, if not higher, than that of equivalently qualified non-Indigenous Australians [12, 13]. Whereas unemployment and lack of job security disproportionately impact those with lower levels of education and those residing in regional areas [18, 25]. With lower levels of educational qualifications the importance of traineeships, certificates and licences (such as driver licences) become critical in securing employment opportunities [12, 18]. Our results support this showing that 63% of those who reported improvements in employment were from regional areas and further clients from regional areas were more likely (OR: 1.72, 95% CI: 1.27–2.33, *p* < 0.001) to obtain an independent licence than clients from urban areas. Recent evidence shows licensing programs are being delivered in regional communities with increased frequency [7]. These regional settings of program delivery provide an opportunity to further improve employment outcomes through facilitating access to employment pathways post licence attainment for unemployed or at-need clients. Pilot studies are needed to inform how this could be applied. One potential opportunity would be for program subsidisation by Service Australia which positions enrolment into licensing programs as a training service for eligible at need job seekers. Another opportunity could be to provide Aboriginal Employment Organisations with the capacity to host and deliver licensing programs within communities. This would enable unemployed clients to be targeted and licensing program staff to be situated within an organisation best placed to secure placement into paid employment post licence attainment.

A key strength of this program leading to translation of outcomes, has been through direct Government collaboration where stakeholders from both Transport for NSW Centre for Road Safety (TfNSW CRS) and Roads and Maritime Services (Aboriginal programs) and representatives from a range of other key agencies formed members of the steering committee, which met quarterly. This allowed the program to be refined via input as it was implemented and importantly also allowed program outcomes and learnings to be fed directly back to government agencies with responsibility for the delivery of such services. This informed development and funding of a disadvantaged Driver Licensing Access Program which delivers services across the State [28], with TfNSW CRS representatives highlighting that this was directly informed by the program. Of the 11 sites that originally participated in the Driving Change program seven sites were awarded funding to continue local program delivery: two urban and three regional sites received funding through Roads and Maritime Services; and another two regional sites received NSW Department of Justice Community Safety Grant funding. It is evident that Government collaboration throughout the program delivery has resulted in meeting key recommendation of the Australian Law Reform Commission that ‘...*State and Territory governments enhance and commit to current government driver education programs, so as to extend the geographic reach of the program and the consistency of service in certain areas*...’ [4]. Opportunities for direct Government collaboration where stakeholders from Department of Education, Skills and Employment or peak body Aboriginal Employment Organisation are members of driver licensing program steering committees may present as initial strategies to establish interagency collaboration between licensing and employment services.

A strength in program data was the inclusion of questions specifically related to employment outcomes which allowed for the programs impact on employment to be directly assessed. Historically no studies have reported licensing programs to deliberately include employment data and outcomes as an evaluable program component [7]. Understanding the types of jobs gained and characteristics of clients gaining employment will allow for greater understanding of the link between driver licensing and employment.

A limitation in program data was the homogeneity in those who participated in providing feedback, as most respondents were secondary educated (or a current secondary student) between the ages of 16–24 years. However, this may not be limited to post program analysis, as respondents’ characteristics were representative of the characteristic of those who did not respondent.

## Conclusion

Driver licensing programs appear to be effective in improving employment outcomes for Aboriginal and Torres Strait Islander clients who obtain a driver licence, and those in regional areas are more likely to obtain a driver licence than those participating in urban areas. Future licensing programs need to consider mechanisms for facilitating employment opportunities post program for clients who successfully obtain a driver licence. Developing such pathways will provide holistic and integrated services to address the social needs of Aboriginal and Torres Strait Islander Australians.

## Data Availability

The datasets used and/or analysed during the current study available from the corresponding author on reasonable request.

## Abbreviations

NSW: New South Wales
ALRC: Australian Law Reform Commission
TfNSW CRS: Transport for NSW Centre for Road Safety
